# Regions of Interest Multivariate Curve Resolution Liquid Chromatography with Data-Independent Acquisition Tandem Mass Spectrometry

**DOI:** 10.1021/acs.analchem.2c05704

**Published:** 2023-05-05

**Authors:** Carlos Pérez-López, Bernat Oró-Nolla, Silvia Lacorte, Romà Tauler

**Affiliations:** Department of Environmental Chemistry, IDAEA-CSIC, Jordi Girona 18-26, Barcelona 08034, Spain

## Abstract

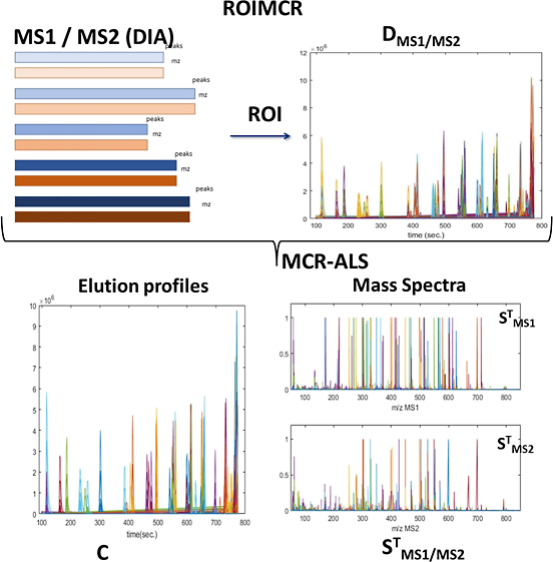

New data-independent acquisition (DIA) modes coupled to chromatographic separations are opening new perspectives in the processing of massive mass spectrometric (MS) data using chemometric methods. In this work, the application of the regions of interest multivariate curve resolution (ROIMCR) method is shown for the simultaneous analysis of MS1 and MS2 DIA raw data obtained by liquid chromatography coupled to quadrupole-time-of-flight MS analysis. The ROIMCR method proposed in this work relies on the intrinsic bilinear structure of the MS1 and MS2 experimental data which allows us for the fast direct resolution of the elution and spectral profiles of all sample constituents giving measurable MS signals, without needing any further data pretreatment such as peak matching, alignment, or modeling. Compound annotation and identification can be achieved directly by the comparison of the ROIMCR-resolved MS1 and MS2 spectra with those from standards or from mass spectral libraries. ROIMCR elution profiles of the resolved components can be used to build calibration curves for the prediction of their concentrations in complex unknown samples. The application of the proposed procedure is shown for the analysis of mixtures of per- and polyfluoroalkyl substances in standard mixtures, spiked hen eggs, and gull egg samples, where these compounds tend to accumulate.

## Introduction

Liquid chromatography (LC) coupled with high-resolution mass spectrometry (HRMS) when preceded by a quadrupole mass analyzer has been shown to be especially suitable for the identification and analysis of chemical compounds in complex environmental samples.^1,2^ Several different approaches have been proposed for this purpose. In the data-dependent acquisition (DDA) mode,^3^ ions are isolated in a narrow *m*/*z* window and individual mass spectra are obtained for the compounds of interest. Alternatively, in data-independent acquisition (DIA) mode,^4^ wider *m*/*z* windows are used to acquire the ions that elute at a given time with the possibility afterward to fragment and collect them all in the same MS2 spectra, like for instance in Bruker broad-band collision-induced dissociation (bbCID) mode.^5^ These MS2 spectra are an alternative to those obtained in DDA modes where only a fixed number of precursor ions are selected and analyzed. There is an intermediate situation where DIA is performed sequentially isolating and fragmenting selected *m*/*z* ranges (e.g., in sequential window acquisition of all theoretical fragment ion spectra, SWATCH^6^). In the analysis of real environmental and biological samples, strong coelutions occur frequently even with optimal chromatographic conditions, making difficult the direct interpretation of the acquired spectra, unless appropriate deconvolution approaches are applied. In this context, different methods for spectral deconvolution have been proposed such as in MS-DIAL,^7−9^ DADIA,^10^ and XCMS.^11,12^

In this work, a completely different perspective and methodology is considered, based on the simultaneous analysis of MS1 and MS2 data acquired by DIA mass fragmentation mode using the regions of interest multivariate curve resolution, ROIMCR, chemometrics method.^13−15^ In this method, two approaches, the ROI and the MCR alternating least squares (MCR-ALS) chemometrics methods,^16,17^ are combined. Whereas the ROI method allows us for a drastic reduction of the size of the huge data sets generated by HRMS instrumentation, filtering the relevant data signals from noise while still preserving their instrumental mass accuracy, the MCR-ALS method allows us for the direct resolution of the elution and spectral profiles of the components of the system which can be then associated with every chemical constituent of the analyzed samples, in a process which can be called component resolution.^18^ In this work, MS1 and MS2 signals (in DIA mode) are analyzed simultaneously by the MCR-ALS,^16,17^ a non-negative matrix factorization method.^19,20^ Surprisingly, as far as we know, no previous work exists at present that takes advantage of this intrinsic bilinear structure of the MS1 and MS2 data and no application has been described about this special structural feature of MS data. This can be especially relevant when the proposed approach is applied for the analysis and compound discovery purposes in complex biological matrices.

In previous works,^13−15^ the ROIMCR method has been applied for gas chromatography (GC)- and LC-HRMS analysis using full-scan MS1 detection only, especially in metabolomics and proteomics studies^21,22^ and more recently for environmental studies.^23,24^ The advantage of using this approach compared to other in the literature like MS-DIAL,^7^ CorrDec,^8^ XCMS,^14^ or metaboanalyst^25^ is precisely that it is based on the concept associated with the bilinear factor decomposition and resolution of the components of a mixture (component resolution) instead of on the concept of individual peak feature selection and deconvolution. In this work, special attention is paid to the bilinear factor decomposition of both MS1 and MS2 data simultaneously (data fusion). Quantitative calibration curves were first built for the prediction of the concentrations of 25 PFAS in standard mixtures and then on spiked and nonspiked bird egg samples.

## Methods

### Samples Analyzed

Three types of samples were analyzed as follows: (a) standard mixture samples of 23 PFAS at 10, 15, 25, 50, 150, and 300 ng/mL; (b) hen eggs spiked with 50 ng of native PFAS; and (c) gull egg samples of the protected species *Larus audouinii* and the scavenger *Larus michahellis* collected in Ebro Delta Natural Park and used as sentinels for PFAS pollution.^26^ To ensure optimal HRMS analysis and enable internal standard quantification, all samples were spiked with 50 ng of a mass-labeled surrogate solution containing 9 MPFAS. The extraction procedure is detailed in a previous study where the performance of the method is well described.^26^ Briefly, 1 g of fresh pooled eggs (12 eggs per pool, to make the sample representative) was extracted with acetonitrile, and the clean-up was performed with activated carbon and glacial acetic acid. This extraction protocol is specific for fluoro-containing chemicals, and contaminants from other chemical families would not be extracted. Further experimental details concerning chemicals and reagents and extraction procedure are given in the Supporting Information.

### UHPLC-qTOF—MS1 and MS2 Analysis

Analysis was performed by LC-quadrupole-time-of-flight (qTOF) (Bruker Impact II Q-TOF mass spectrometer) and data acquisition conditions (full-scan mode at a mass range from 30 to 1000 *m*/*z* and DIA obtained through a 6 eV energy for MS1 and through a 30 eV energy for MS2 using bbCID from Bruker technology). bbCID generates an extensive data set by independently acquiring continuous high-resolution accurate mass precursor ions’, true isotope patterns or fragment qualifier ions throughout the analysis. This way of working allows obtaining fragments at two different selected energies. This unbiased approach, with an elevated collision energy and no isolation of specific precursor ions during bbCID data acquisition, provides analytical advantages such as the following: (i) discrimination capacity for the detection of an unlimited number of compounds over a large dynamic range; (ii) retrospective screening and discovery workflows; (iii) suspect compound screening based on accurate mass determination and isotope pattern fit, without the need of reference standards; and (iv) possible detection of trace compounds that, due to their low intensity, would not be detected in DDA mode.

### Chemometric Methods

**Figure 1:**
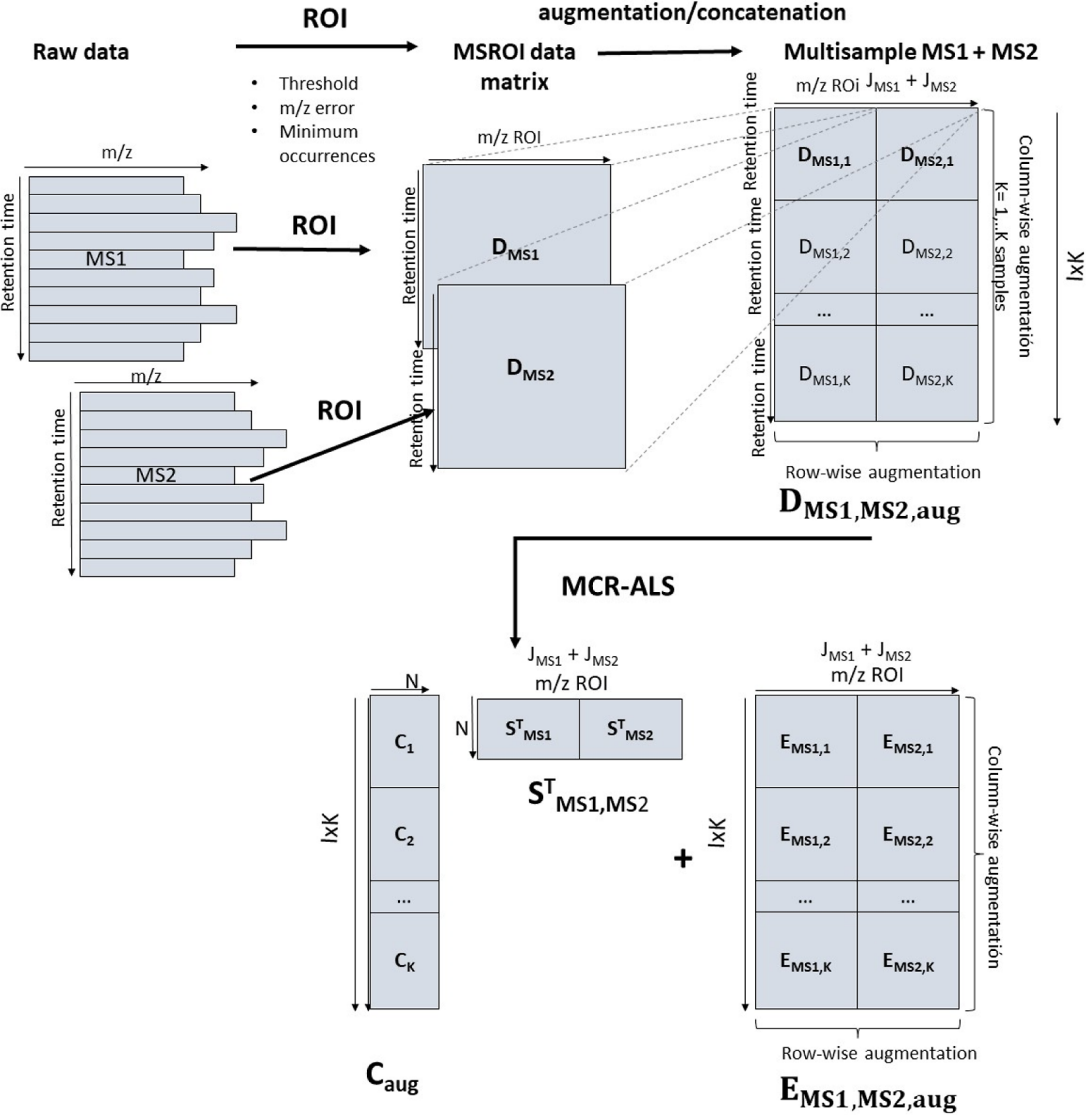
Building, augmenting, and concatenating of MSROI data matrices and MCR-ALS bilinear modeling.

The ROIMCR chemometric method proposed in this work has two independent parts, the selection of the ROI and the application of the MCR-ALS method (see Figure 1).

### ROI Method

The selection of the ROI of LC–MS raw data has been described in previous works.^13−15^ ROI methodology searches and selects the significant HRMS signals obtained in the analysis of every sample (chromatographic run) and builds the corresponding MSROI data matrices. The ROI method scans the mass spectra for regions where (1) the ion signal is above the selected threshold (above the instrumental noise), (2) the masses agree within a present mass accuracy tolerance, and (3) the masses occur repeatedly over a time range consistent with the expected width of a chromatographic peak. This selected time range should be consistent with the instrument setup (e.g., GC or LC) and the type of chromatographic column. ROI methodology results in a significant reduction of the computer storage and resources, and it keeps the mass accuracy of the original HRMS measurements. The ROI method defines what signals should be considered in the subsequent data analysis and resolution. The number of ROIs should include all the significant features related to the composition of the sample. ROIs can be selected for any set of samples under different experimentally designed conditions or simultaneously for the whole set of samples to be analyzed, for instance in quantitative determinations for calibration and unknown samples. In any case, the application of the ROI procedure will deliver a data matrix, where (see Figure 1) the rows are the mass spectra acquired at the considered retention times and the columns are the ion chromatograms acquired at the selected ROI *m*/*z* values. This is valid for the analysis of a single data matrix in the case of a single chromatographic run or sample (***D***_**MS1**_ and ***D***_**MS2**_) and for the analysis of a column-wise data matrix in the simultaneous analysis of multiple chromatographic runs of several samples. In the last case, the data matrices of the individual chromatographic runs are concatenated vertically, as shown in Figure 1. As also shown in this figure, this situation is valid for both MS1 and MS2 acquisition modes. Since in both cases, ***D***_**MS1**_ and ***D***_**MS2**_, the rows (retention times) of these two matrices are the same, their horizontal concatenation is possible, giving a row-wise augmented data matrix. In the case of multiple chromatographic runs simultaneously analyzed, a row- and column-wise super augmented data matrix with both types of MS signals (MS1 and MS2) and for all the samples simultaneously analyzed (see Figure 1) is obtained, ***D***_**MS1,MS2,aug**_. This type of MS1 and MS2 ROI augmentation and simultaneous analysis is proposed in this work for the first time. Once the ROI data matrices have been arranged properly for the case of the study, they can be analyzed by the MCR-ALS method.

### MCR-ALS Method

The MCR-ALS method has been previously described in detail.^16,17^ Basically, it performs the bilinear factor decomposition of the experimental data matrix according to the matrix eq 1
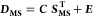
1where ***D***_**MS**_ (*I*×*J*) is the MS ROI experimental data matrix arranged as described above, (see Figure 1), with *I* rows (number of retention times and mass scans) and *J* columns (number of mass traces and *m*/*z* values), and ***C***(*I*×*N*) and ***S***_**MS**_^**T**^(*N*×*J*) are the two factor matrices obtained in the bilinear decomposition for a number of components *N*, with their resolved elution (concentration) profiles and their resolved mass spectra. The mass spectra resolved in ***S***_**MS**_^**T**^ have the ion signals of the different components resolved by MCR-ALS which can be then used for annotation and identification purposes, i.e., to discover what chemical compound is associated with this signal. On the other hand, the elution profiles of the different components resolved in the ***C*** matrix can also be associated with the concentration of the different chemical constituents in the analyzed sample. Finally, in eq 1, ***E*** refers to the unexplained experimental data variation or data residuals. Ideally, these residuals should not contain the sought signals of the chemical constituents of the samples.

Equation 1 is valid for MS1 and MS2 data sets (see Figure 1). In the case of MS1, ***S***_**MS1**_^**T**^ will only contain the signals of the precursor ions and of some low-energy fragments and adducts present in the signals. In contrast, for MS2, ***S***_**MS2**_^**T**^ will contain the signals of the fragments obtained at the higher collision energies used. These MS1 and MS2 spectra provide the information needed to identify and characterize the sample chemical constituents.

The simple bilinear model described by eq 1 can be easily extended to the simultaneous analysis of multiple (*K*) chromatographic runs and to the MS1 and MS2 acquisition modes, as shown in Figure 1 and eq 2.
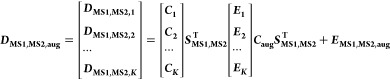
2where ***C***_**aug**_(*IK*,*N*) has now the elution profiles of the *N*-resolved components in the *K* samples/chromatographic runs simultaneously analyzed. From these profiles, their peak areas and maximum heights are easily calculated, and their relative concentrations are derived (see below). ***S***_**MS1,MS2**_^**T**^ has now the mass spectra of the *N*-resolved components in the two acquisition modes, MS1 and MS2, concatenated horizontally.

The more interesting advantage of the bilinear decomposition proposed by eq 2 is that every resolved component will have the MS1 and MS2 signals in  and therefore that they can be used directly for compound identification. In addition to the possibilities of achieving an improved resolution and increasing the sensitivity of analysis, the quantitation of the sample constituents will also increase because the information provided by the two acquisition modes in  is complementary and facilitates their unique identification and quantitative determination in . MCR-ALS^16,17^ solves bilinear eqs 1 and 2 by minimization of the sum of the squares of the residuals (different ***E*** terms in eqs 1 and 2), having the data variance not explained by the MCR bilinear model.

Inherent uncertainties associated to MCR resolution are low in the case of the analysis of LC-HRMS data due to the inherent sparsity nature of MS spectra which have many 0 values and high selectivity.^13−17^ Only in the case of total coelution and/or equal MS signals, resolution uncertainties are high due to rotation ambiguities. One special case would be the ubiquitous presence of solvent MS signals appearing in the resolved spectra of the sample constituents if they are not eliminated, either before or after the ROIMCR analysis.

MCR-ALS was applied to ***D***_**MS1,aug**_, ***D***_**MS2,aug**_, and to ***D***_**MS1/MS2,aug**_ (see Figure 1). Further details about the MCR-ALS minimization procedure are given in the Supporting Information and in previous works^16,17^

### Identification of Sample Constituents from the ROIMCR-Resolved Elution and Spectral Profiles

The results obtained by the ROIMCR analysis of the data described above provide the mass spectra (in ***S***_**MS**_^**T**^) and elution profiles (in ***C***) of the resolved components (see eqs 1 and 2). The spectral information (either in ***S***_**MS1**_^**T**^, ***S***_**MS2**_^**T**^, or ***S***_**MS1,MS2**_^**T**^ matrices) and the retention times at the peak maxima of the elution profiles (in ***C*** matrix) can be used for the identification of the chemical compounds associated with them. This approach differs from other approaches recently proposed^3,4,6−12^ based on the extraction of individual signal features, which require their further processing and deconvolution in different consecutive steps, such as peak alignment, peak matching, peak modeling, and peak feature correlation. None of these steps are needed in the proposed ROIMCR approach, which is obviously a remarkable advantage.

Ion information in ***S***_**MS1**_^**T**^, ***S***_**MS2**_^**T**^, or ***S***_**MS1,MS2**_^**T**^ matrices (in MS1 and MS2) is matched with the corresponding *m*/*z* values of previously known compounds (i.e., standards) or with those stored and compiled in public databases such as in the Human Metabolome Database (HMDB,^30^), PubChem,^31^ MassBank,^32^ or MoNA.^33^ The analysis of the fragmentation pattern of every sample constituent in the case of the MS2 part of  spectra is linked and associated directly to the information about the precursor ions obtained in the MS1 spectrum. In contrast to the existing methodologies which are based on the individual separated extraction of signal features either in MS1 or in MS2 raw experimental data sets, the proposed procedure represents a step forward in the compound identification/annotation steps because the link between the precursor and fragment ions is directly obtained and visualized, without any further data post processing.

### Calibration/Quantitation/Recovery of Sample Constituents Using Peak Heights of the ROIMCR-Resolved Elution Profiles

ROIMCR simultaneous analysis of multiple samples provides rich information about the relative amounts of their chemical constituents (in the ***C*** or ***C***_**aug**_ matrices of eqs 1 and 2). The peak heights of the ROIMCR-resolved elution profiles of every component in the different samples can be used for the relative quantification of the chemical compounds associated with these components in the analyzed samples. Calibration curves can be built using the known concentrations in the simultaneously analyzed standard mixture. The quality of the calibration equations can be then assessed by the comparison between predicted and actual (known) concentrations, with regression lines whose slopes should be close to 1, offsets close to 0, and correlation coefficients close to 1. The root-mean-square error values in the calibration sample (RMSEC) values and the mean relative errors (RE %) can also be estimated to assess the quality of the calibration procedure.
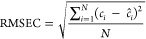
3where *c*_*i*_ and  are, respectively, the nominal and the predicted concentrations of every PFAS in the N standard mixture samples.
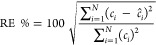
4

Internal standard calibration curves were built using the information of the peak heights and concentration of the PFAS and surrogates (MPFAS) in the standard mixtures. Peak heights and nominal concentrations of the calibration standards were divided by the peak heights and nominal concentrations of the surrogates. Finally, to determine the extraction efficiency and accuracy, the compound recoveries (% *R*) were calculated. For this purpose, hen egg samples spiked with known concentrations of PFAS were extracted using the same sample pretreatment and clean-up procedures^26^ and the analyte concentrations were estimated using previously developed calibration curves. The estimated concentrations were then compared to the known concentrations in these spiked hen egg samples, and the recovery percentages were estimated.

## Results and Discussion

### LC–MS1, LC–MS2, and LC–MS1/MS2 Analysis of PFAS Samples

**Figure 2:**
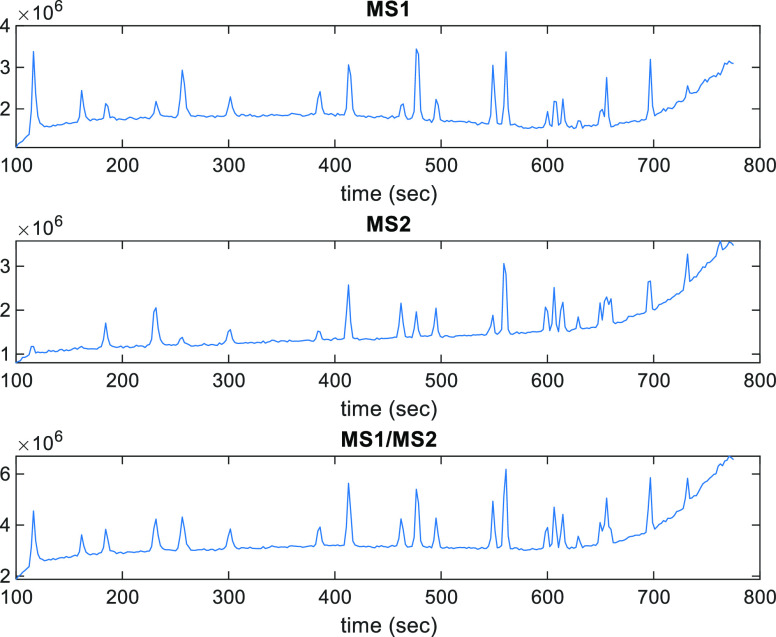
TIC chromatograms (summed TIC) of the 50 ng/mL standard PFAS mixture obtained by MS1 (upper), MS2 (middle), and MS1/MS2 DIA sum (lower). See Figure 3 for their, respective, MCR-ALS resolution results.

As an example of data experimentally acquired, the total ion current (TIC) LC-qTOF-MS chromatograms of the standard PFAS mixture at 50 ng/mL using MS1, MS2, and summed MS1/M2 DIA are given in Figure 2. Elution patterns observed in the TIC obtained by these three different detection modes show some differences, specially between MS1 and MS2 signals due to their different ion sensitivities. The advantage of using the MS1/MS2 joint DIA detection is that these patterns are summarized in a single chromatogram, and therefore, their joint analysis is feasible and captures all the features observed by these two detection systems, MS1 and MS2. This allows us also for an improved resolution, identification, and quantitation of the chemical constituents present in the analyzed samples. In Figure S1, the TIC and TIMS (total ion mass spectra) of one of the spiked hen egg samples and of one of the gull egg samples are given for the case of the MS1/MS2 simultaneous detection. Some differences are appreciated between the TIC of these two samples and with the TIC and TIMS of the mixture standard sample shown previously in Figure 2.

### ROIMCR Resolution and Identification of PFAS in the Analyzed Samples

The ROI compression/filtering procedure was applied to the different MS1 and MS2 data sets analyzed in this work corresponding to the analysis of the PFAS standard mixture samples at 10, 15, 25, 50, 150, and 300 ng/mL, spiked hen egg, and gull egg samples. The selected values for the ROI parameters were the same for the three MS detection approaches (signal intensity threshold = 1100, *m*/*z* deviation = 0.005, and minimum number of occurrences per chromatographic run = 5). The resulting number of ROIs for individual MS1 and MS2 data matrices (single chromatographic runs) varied between 1300 and 1400 and between 900 and 1000, respectively. In the simultaneous analysis of all data sets and samples, the numbers of ROIs were 3355 for MS1 (in ***D***_**MS1,aug**_) and 2249 for MS2 (in ***D***_**MS2,aug**_). These ROIs include the MS signals (features) associated with the chemical constituents of the samples and with some additional MS signals associated with background, solvent and other instrumental contributions. In the case of the super-augmented data matrix, ***D***_**MS1,MS2,aug**_, the total final number of ROIs was 5604 (the sum of those obtained for the augmented data matrices ***D***_**MS1,aug**_ and ***D***_**MS2,aug**_). ROI data matrices require less computer storage and shorter processing times compared to the raw data, without losing their original instrumental mass accuracy.

After ROI data processing, MCR-ALS was applied to the different types of augmented MS data matrices, ***D***_**MS1,aug**_, ***D***_**MS2,aug**_, and to ***D***_**MS1/MS2,aug**_. The number of MCR-ALS components (NC) and the amount of data variances explained (*R*^2^) in these three cases were, respectively, ***D***_**MS1,aug**_, NC = 50, *R*^2^ = 99.3%, ***D***_**MS2,aug**_, NC = 55, *R*^2^ = 99.3%, and ***D***_**MS1/MS2,aug**_, NC = 55, *R*^2^ = 96.4%. When needed, spurious minor MS signals in the resolved components were eliminated by application of a lower threshold value constraint during the ALS optimization.^16,17^ Resolved elution and spectral profiles showed good chromatographic and spectral features, except for those related to background, solvent, or noise instrumental signals. In the case of the simultaneous analysis of the ***D***_**MS1/MS2,aug**_ augmented data matrix, a slightly worse data fit was obtained due to small peak alignment matching disagreements between the two types of MS signals, MS1 and MS2.

**Figure 3:**
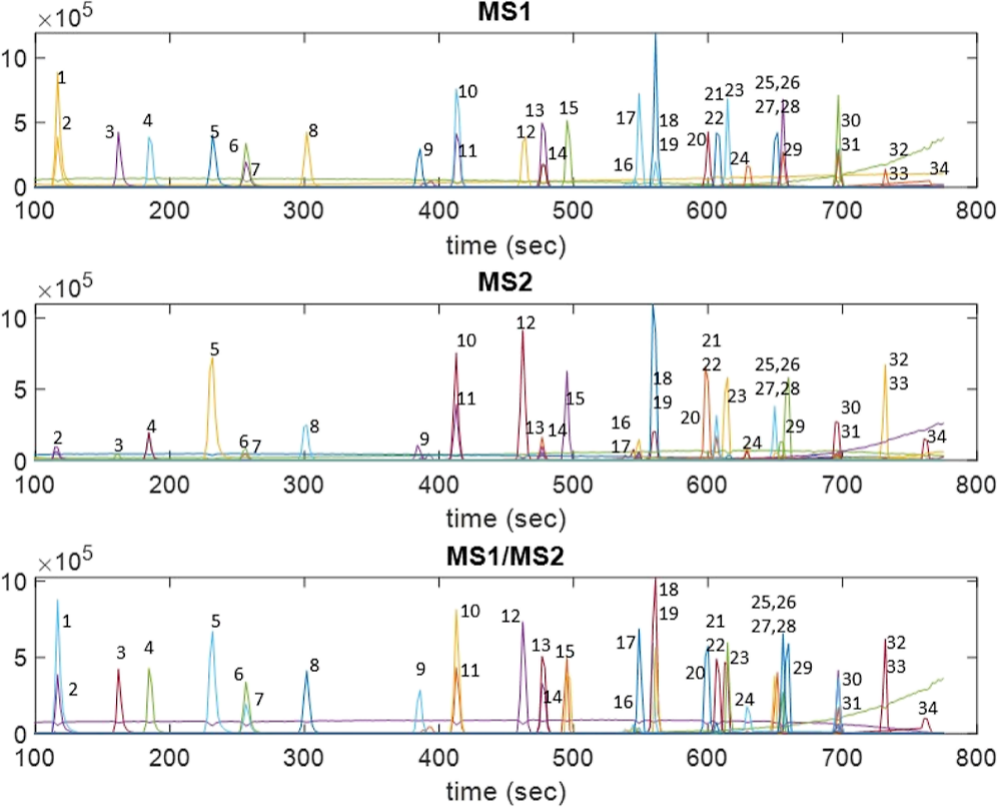
ROIMCR-resolved elution profiles in the analysis of ***D***_**MS1,aug**_ (upper), ***D***_**MS2,aug**_ (middle), and to ***D***_**MS1/MS2,aug**_ (lower) for the 50 ng/mL standard PFAS mixture sample (see also their TIC on Figure 2). Elution profiles are identified by a number which corresponds to the ROIMCR components listed given in Table 1 and identified as PFAS in Table S1.

In Figure 3, an example of the results of the application of ROIMCR to MS1 data (***D***_**MS1,aug**_), MS2 data (***D***_**MS2,aug**_), and MS1/MS2 data (***D***_**MS1/MS2,aug**_) is shown in the analysis of the 50 ng/mL PFAS standard mixture. This example is used to show the possibilities of the proposed ROIMCR method. The two subplots in the upper and middle parts of Figure 3 give the resolved elution profiles in the separate ROIMCR analysis of MS1 (***D***_**MS1,aug**_) and MS2 (***D***_**MS2,aug**_) data matrices. Observe that some of the elution profiles were not clearly resolved or gave very little signal when only the MS2 data were analyzed. The results of the simultaneous analysis of MS1 and MS2 data (***D***_**MS1/MS2,aug**_) (in the lowest part of Figure 3) summarize better the resolved elution profiles of the different sample constituents using these two MS detection modes. ROIMCR-resolved components were annotated and identified in the three subplots using the retention times of the elution profiles at their peak maxima and the *m*/*z* values of the more intense ion signals in the corresponding MS1 and MS2 spectra which were compared with their nominal values (see below and Table S1). In a few cases, the annotation of the elution profiles was difficult due to the coincidence of the elution profiles of PFAS with those of their surrogates (peaks 1 and 2, 6 and 7, 10 and 11, 13 and 14, 16 and 17, 18 and 19, 21 and 22, 26 and 27, and 30 and 31, also in Tables 1 and S1). In these cases, if needed, the MCRALS results of their time sub windows were analyzed in detail and their elution profiles were finally resolved. These results were compared with those obtained using the Bruker Data Analysis^5^ approach for the same standard mixture at 50 μg/L. The coincidence between the results of the two approaches in the analysis of this PFAS mixture standard was excellent (see Figure S2 and its text explanation).

**Table 1:** PFAS Internal Standard Calibration Parameters in the Standard Mixture Samples and Recoveries (%R) in the Spiked Hen Egg Samples^a^*

		MS1^1^				MS2^2^				MS1/MS2^3^				% *R*
peak Nr	PFAS	*r*^2^	RMSEC	RE %	*N* (ug/L)	*r*^2^	RMSEC	RE %	*N* (ug/L)	*r*^2^	RMSEC	RE %	*N* (ug/L)	%
1, 2	PFBA, MPBA	0.9922	0.2	9.1	5 (40–600)	0.9984	0.1	4.1	5 (40–600)	0.9920	0.2	9.1	5 (40–600)	77
3	PFPeA	0.9917	0.1	9.4	5 (20–300)	0.9834	0.2	13.3	5 (20–300)	0.9914	0.1	9.5	5 (20–300)	71
4	L-PFBS**	0.9910	0.1	9.7	5 (10–150)	0.9914	1.0	6.9	4 (10–50)	0.9924	0.1	8.9	5 (10–150)	55
5	4:2 FTS	0.9989	0.1	3.4	5 (40–600)	0.9769	0.7	16.4	6 (40–1200)	0.9949	0.2	7.3	5 (40–600)	72
6, 7	PFHxA, MPFHxA	0.9906	0.1	10.0	5 (10–150)	0.9888	0.1	10.9	5 (10–150)	0.9904	0.1	10.1	5 (10–150)	78
8	L-PFPeS	0.9943	0.0	7.7	5 (10–150)	0.9940	0.1	7.9	5 (10–150)	0.9864	0.1	12.0	5 (10–150)	73
9	PFHpA	0.9941	0.1	7.8	5 (10–150)	0.9902	0.1	10.2	5 (10–150)	0.9942	0.1	7.8	5 (10–150)	76
10, 11	Mbr-PFHxS, br-PFHxS	0.9858	0.1	12.2	5 (10–150)	0.9946	0.0	7.5	5 (10–150)	0.9896	0.1	10.5	5 (10–150)	81
12	6:2 FTS	0.9939	0.2	8.0	5 (40–600)	0.9992	0.1	2.7	5 (60–600)	0.9974	0.1	5.2	5 (40–600)	82
13, 14	PFOA**, MPFOA	0.9760	0.2	15.7	5 (15–300)	0.9962	3.8	5.2	6 (10–300)	0.9891	0.1	10.5	5 (15–300)	54
15	L-PFHpS	0.9908	0.1	9.8	5 (10–150)	0.9972	0.0	5.4	5 (10–150)	0.9934	0.1	8.3	5 (10–150)	83
16, 17	MPFNA, PFNA**	0.9895	0.1	10.5	5 (10–150)	0.9973	3.1	4.4	6 (10–300)	0.9936	0.1	7.6	4 (15–150)	63
18, 19	Mbr-PFOSK Br-PFOSK	0.9923	0.1	9.0	5 (10–150)	0.9939	0.1	8.0	5 (10–150)	0.9810	0.2	14.0	5 (15–300)	91
20	8:2 FTS	0.9984	0.1	4.0	5 (40–600)	0.9945	0.2	7.6	5 (40–600)	0.9947	0.4	7.8	6 (40–1200)	75
21, 22	PFDA, MPFDA	0.9794	0.1	13.7	4 (15–150)	0.9849	0.2	12.4	5 (15–300)	0.9904	0.1	9.8	5 (15–300)	65
23	L-PFNS	0.9974	0.0	5.2	5 (10–150)	0.9887	0.1	11.4	6 (10–300)	0.9878	0.2	11.1	5 (15–300)	83
24	br--*N*MeFOSAA	0.9921	0.1	8.4	4 (15–150)	0.9823	0.2	14.3	6 (10–300)	0.9904	0.2	8.9	4 (15–150)	73
25	FOSA	0.9947	0.0	7.4	5 (10–150)	0.9733	0.2	16.6	5 (15–300)	0.9996	0.0	2.1	5 (10–150)	63
26, 27, 28	PFUdA***, MPFUdA, br-*N*-EtFOSAA									0.9904	0.1	10.0	6 (10–300)	95
30,31	PFDoA**, MPFDoA*	0.9940	0.1	7.9	5 (10–150)	0.9926	5.2	7.3	6 (10–300)	0.9782	0.2	15.0	5 (15–300)	64
32,33	PFTrDA***, L-PFDoS									0.9821	0.2	12.2	4 (25–300)	72
34	PFTeDA**	0.994	0.1	7.4	4 (15–150)	0.9912	5.7	10.0	6 (10–300)	0.9940	0.1	7.9	5 (10–150)	64

a *br-N-EtFOSAA and L-PFDoS were not properly resolved and not shown in the table. ** MS2 calibration parameters were obtained using external standard calibration. *** Resolution of elution profiles was only possible in the analysis of MS1/MS2 data. Calibration results using only MS1 signals1^1^ and using only MS2 signals^2^. Also, using MS1/MS2 signals^3^: *r*^2^, correlation coefficients of the regression lines between calculated and actual PFAS concentrations, root-mean-square error in PFAS calibration, RMSEC= , relative error in the prediction of PFAS, RE% = 100 , where *c*_*i*_ and  are, respectively, the correct and the predicted concentrations of every PFAS in the N standard mixture samples N, number of samples in the regression lines and in parenthesis PFAS concentration interval in micrograms per liter. ^4^PFAS recoveries in spiked hen eggs samples.

Elution profiles resolved in the separate analysis of hen egg samples on one side and of gull egg samples on the other side, without considering PFAS standard mixture samples when both MS1 and MS2 signals were simultaneously analyzed, are given in Figure S3. In all analyzed samples, the elution profiles of the native compounds (in spiked hen egg samples) and surrogates (in gull egg samples) were properly resolved (see peak numbers and their identification in Table S1). Compared to PFAS standard mixture samples, in the case of spiked hen egg and gull egg samples, some additional chemical compounds (peaks 35–40) were resolved that elute at the highest retention times.

The details of the results obtained in the identification/annotation of all the different compounds present in the analyzed samples using the proposed ROIMCR approach are given in Table S1. Most of the MS2 spectra of the PFAS compounds were properly resolved and identified in the standard mixture and spiked hen egg samples, except for br-*N*-EtFOSAA and L-PFDoS which could not be properly resolved because they totally coeluted with PFUdA and PFTrDA at 655.7 and 731.9 s, respectively (see Table S1). The retention times and major MS1 and MS2 signals of all ROIMCR components are given in the table. Two additional compounds were lipids identified as palmitoleic and docosahexaenoic acids, which were present in the hen and gull egg samples. The other four components (peaks 37–40), found only in gull egg samples, gave well-defined MS1 and MS2 spectral profiles (see table values), but they were not properly identified using available MS databases (MoNA, MassBank of North America, https://mona.fiehnlab.ucdavis.edu/ and **MassBank, https://massbank.eu/MassBank/).

**Figure 4:**
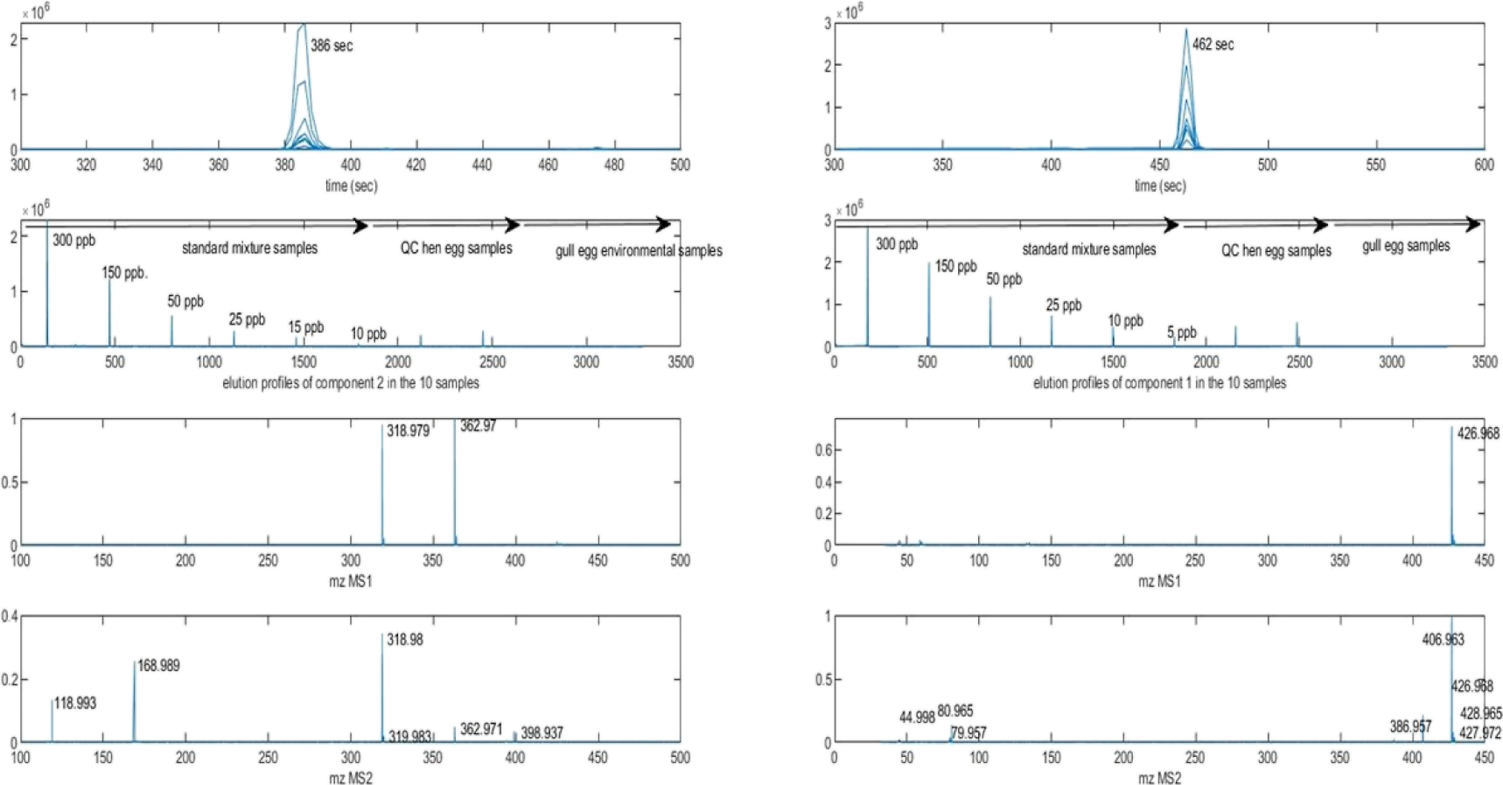
Resolution of ROIMCR components nr 9 (left) and 12 (right) identified, respectively, as PFHpA and 6:2 FTS (see Tables 1 and S1). In the upper subplot, elution profiles resolved in the different samples are overlaid with their peak maxima at 386 and 462 s (6.4 and 7.7 min), respectively. Below, the same elution profiles are plotted sequentially, in the six standard mixture samples at the PFAS concentrations 10, 15, 25, 50, 150, and 300 ng/mL, in the two spiked hen eggs at 50 ng/g, and in the two gull egg samples. In the two subplots given below, the ROIMCR resolved ***S***_**MS1**_^**T**^ (precursor ions) and ***S***_**MS2**_^**T**^ (fragment ions) spectra, with the *m*/*z* values of the ion signals of higher intensity.

Figure 4 shows graphically two examples of the results obtained in the resolution of two sample constituents directly linking their elution profiles with their corresponding MS1 and MS2 mass spectra, which facilitates their identification. The resolved elution and spectral profiles for the ROIMCR components numbers 9 (left) and 12 (right) identified as PFHpA and 6:2 FTS (see Tables 1 and S1) are shown. In the upper part of Figure 4, the elution profiles of these components in the different samples are overlaid, with their peak maxima at the retention times of 386 and 462 s (6.4 and 7.7 min), respectively. In the middle of the figure, the same elution profiles are plotted sequentially for all the different samples simultaneously analyzed (in ***D***_**MS1/MS2,aug**_ data matrix). The first six profiles are at the concentrations 10, 15, 25, 50, and 150 to 300 ng/mL. The peak heights of these profiles were used to build their calibration curves and derive quantitative information (see below). The two next elution profiles correspond to the two spiked hen egg samples spiked at 50 ng/g. Observe that the peak heights of these two elution profiles are lower than those of the same concentration in the standard mixture sample at 50 ng/mL This difference was used to evaluate the recovery of these two compounds (76 and 82%, see below in Table 1) from the peak areas of their elution profiles resolved by ROIMCR in the spiked hen egg samples. These two PFAS were not detected however at significant concentrations in the gull egg samples since no elution profile was resolved in these samples. In the two lowest parts of Figure 4, the MS1 and MS2 spectra of this ROIMCR component are given separately, showing their ion signals at *m*/*z* values of 362.97 and 318.98 (left) and 118.99 (right) in MS1 and at 319.98, 168.99, and 118.99 (left) and 80.96, 406.96, 426.97, and 427.97 (right) in MS2 (see Table S1). All this information was used to identify these two ROIMCR components as PFHpA and 6:2 FTS. Similar assignments were performed for the rest of the other ROIMCR-resolved components which are finally summarized in Table S1.

### ROIMCR Calibration and Quantitative Analysis of PFAS in the Analyzed Samples

Peak heights of the different ROIMCR-resolved components (PFAS and surrogates) in the standard mixture using individual MS1 and MS2 signals on one side and using simultaneously MS1/MS2 signals on the other side were used to establish internal standard calibration curves for each compound using their corresponding surrogates. Results are summarized in Table 1. Peaks of every PFAS and surrogate are identified (first column) by a number which is the same as that given in Figures 3 and S3 and with their retention times in seconds. Correlation coefficients, *r*^2^, between the concentrations predicted of the PFAS in the calibration samples and the correct concentration values, root-mean-square errors in calibration, RMSEC (eq 3), and the relative errors, RE (eq 4), are given for the three calibration cases, as well as the number of standards (*n*) used and their concentration range (μg/L) used to build the calibration curves. In many cases, RE values were lower than 10%. All regression coefficients were close to 1, which means that the predicted concentrations were close to the correct ones. MS1/MS2 calibration results were usually intermediate or even better than those obtained using either MS1 or MS2 separately. Only in few cases, the MS2 internal calibration method was not possible because of the difficulty in resolving the native and surrogate standards (similar fragment ions formed). In these cases (marked by * in Table 1), results were obtained using the external calibration approach (without using surrogates). The calculation of the PFAS concentrations in the spiked hen egg samples allowed us for the estimation of their concentrations and of their recoveries (see the last column of Table 1). In most of the cases, the PFAS recoveries obtained in the spiked hen egg samples were reasonably good, always above 50%, with an average recovery around 75%.

In addition, quantitation of PFAS compounds encountered in the unknown gull egg samples using the ROIMCR-developed calibration curves was possible. In a previous study, the most prominent PFAS in gulls was found to be b**r-**PFOSK.^26^ As an example, the concentrations of br-PFOSK and br-PFHxS (see Table1), also encountered in these two samples, were estimated, respectively, to be 35 and 11.5 ng/g wet weight in the *L. audouinii* gull egg sample and 36 and 11.5 ng/g wet weight in the *L. michahellis* gull egg sample (see samples analyzed in Figure S3). Other PFAS encountered at very low concentrations in these two gull egg samples could not be quantified in a reliable way.

## Conclusions

The new DIA modes in HPLC-qTOF MS facilitate simultaneous acquisition of full-scan MS1 and MS2 data. The ROIMCR approach offers direct resolution of the chemical constituents in environmental samples, preserving mass accuracy and enabling easy identification and annotation, linking MS1 precursor ions with their MS2 fragments. It eliminates the need for peak retention time alignment, peak matching, and background correction and reduces difficulties in data analysis. MS1 and MS2 calibration and quantitation are also simplified. These advantages were demonstrated in the analysis of 23 PFAS in spiked hen and gull egg samples. The proposed ROIMCR approach can be generalized for compound discovery, identification, and quantitation in environmental, food, health, and omics (metabolomics and lipidomics) samples.

## Supporting Information Available

Description of experimental methods: chemicals, reagents, and extraction procedure; UHPLC-qTOF—MS1 and MS2 analysis; short description of the MCR-ALS method; identification of ROIMCR components and its detailed explanation; MS1/MS2 sum TIC and TIMS of one spiked hen egg and of one gull egg sample; elution profiles obtained by compass data analysis (Bruker Scientific) of the 50 ng/mL standard PFAS mixture sample by MS1 (upper), MS2 (middle), and MS1/MS2 DIA (lower); and ROIMCR-resolved elution profiles in the analysis of hen egg and gull egg samples by MS1/MS2 DIA (PDF)
