# Supplementary material for: Regions of Interest Multivariate Curve Resolution Liquid Chromatography with Data-Independent Acquisition Tandem Mass Spectrometry
Source: Anal Chem. 2023 May 5;95(19):7519–27. doi: 10.1021/acs.analchem.2c05704 (PMC10193361; doi:10.1021/acs.analchem.2c05704)
Supplement: Supplementary file 1 — ac2c05704_si_001.pdf [file ac2c05704_si_001.pdf]

Supporting Information

Regions of Interest Multivariate Curve Resolution (ROIMCR) Liquid Chromatography with Data Independent Acquisition Tandem Mass Spectrometry

Carlos Pérez-López[a], Bernat Oró-Nolla[a], Silvia Lacorte[a] and Romà Tauler[a*]

[a] Department of Environmental Chemistry, IDAEA-CSIC. Jordi Girona 18-26, 08034 Barcelona, Spain

* corresponding author: email: Roma.Tauler@idaea.csic.es

Table of contents

1) Description of experimental methods: chemicals, reagents and extraction procedure; UHPLC-qTOF – MS1 and MS2 analysis.

2) Short description of MCR-ALS method.

3) Supporting Table 1. Identification of ROIMCR components and its detailed explanation.

4) Supporting Figure 1. MS1/MS2 Sum TIC and TIMS of one hen egg and of one gull egg sample

5) Supporting Figure 2. Elution profiles obtained by Compass Data Analysis (Bruker Scientific) of the 50 ng/mL standard PFAS mixture sample by MS1, MS2 and MS1/MS2 DIA

6) Supporting Figure 3. ROIMCR resolved elution profiles in the analysis of hen egg and gull egg samples by MS1/MS2 DIA

DESCRIPTION OF EXPERIMENTAL METHODS

Chemicals and reagents

PFAC-MXH native multi concentration mixture solution was acquired from Wellington Laboratories (Guelph, Ontario, Canada) and contained perfluoro-n-butanoic acid (PFBA) at 4000 ng/mL, perfluoro-n-pentanoic acid (PFPeA) at 2000 ng/mL, perfluoro-n-hexanoic acid (PFHxA), perfluoro-n-heptanoic acid (PFHpA), perfluoro-n-octanoic acid (PFOA), perfluoro-n-nonanoic acid (PFNA), perfluoro-n-decanoic acid (PFDA), perfluoro-n-undecanoic acid (PFUdA), perfluoro-n-dodecanoic acid (PFDoA), perfluoro-n-tridecanoic acid (PFTrDA), perfluoro-n-tetradecanoic acid (PFTeDA), N-methylperfluorooctanesulfonamidoacetic acid (linear and branched isomers) (br-NMeFOSSA), N-ethylperfluorooctanesulfonamidoacetic acid (linear and branched isomers) (br-NEtFOSSA), potassium perfluoro-1-butanesulfonate (L-PFBS), sodium perfluoro-1-pentanesulfonate (L-PFPeS), potassium perfluorohexanesulfonate (linear and branched isomers) (br-PFHxS), sodium perfluoro-1-heptanesulfonate (L-PFHpS), potassium perfluorooctanesulfonate (linear and branched isomers) (br-PFOS), sodium perfluoro-1-nonanesulfonate (L-PFNS), sodium perfluoro-1-decanesulfonate (L-PFDS) and sodium perfluoro-1-dodecanesulfonate (L-PFDoS) at 1000 ng/mL and sodium 1H,1H,2H,2H-perfluorohexanesulfonate (4:2FTS), sodium 1H,1H,2H,2H-perfluorohexanesulfonate (6:2FTS), sodium 1H,1H,2H,2H-perfluorohexanesulfonate (8:2FTS) at 4000 ng/mL. In addition, MPFAC-MXA were used as a surrogate mixture solution and contained 9 isotopic labelled standards at 2000 ng/mL: perfluoro-n-[1,2,3,4-$^{13}C_4$]butanoic acid (MPFBA), perfluoro-n-[1,2-$^{13}C_2$]hexanoic acid (MPFHxA), perfluoro-n-[1,2,3,4-$^{13}C_4$]octanoic acid (MPFOA), perfluoro-n-[1,2,3,4,5-$^{13}C_5$]nonanoic acid (MPFNA), perfluoro-n-[1,2-$^{13}C_2$]decanoic acid (MPFDA), perfluoro-n-[1,2-$^{13}C_2$]undecanoic acid (MPFUdA), perfluoro-n-[1,2-$^{13}C_2$]dodecanoic acid (MPFDoA), sodium perfluoro-1-hexane[$^{18}O_2$]sulfonate (MPFOS), and sodium perfluoro-1-[1,2,3,4-$^{13}C_4$]octanesulfonate (MPFOS). This surrogate mixture solution was added to all samples analyzed.

Chromatography grade acetonitrile (ACN), methanol (MeOH) water, and ammonium acetate (NH_4Ac) were acquired from Merck (Darmstadt, Germany).

Extraction procedure

Sample extraction procedures are given in previous work (Colomer-Vidal P.; Bertolero, A.; Alcaraz, C.; Garreta-Lara, E.; Santos, F.J.; Lacorte, S. Distribution and ten-year temporal trends (2009–2018) of perfluoroalkyl substances in gull eggs from Spanish

breeding colonies, *Environ. Pollut.* **2022**, *293* https://doi.org/10.1016/j.envpol.2021.118555). The protocol used for the analysis of PFAS is specific for these compounds which encompasses a large family of F-containing substances. After an extraction with acetonitrile (not methanol which would extract a much larger amounts of lipids), a cleanup was performed with activated carbon which eliminates the lipids while PFAS are recovered in a theoretically fat-free extract. However, the clean-up is never 100% efficient and some lipids may remain in the final tissue, as visually observed as the extracts are yellow-colored. The analysis by HRMS detects “everything” with high sensitivity, so part of these co-extracted lipids elutes at the very end of the chromatogram (in agreement with their somewhat apolar nature). Therefore, the identification of these additional compounds (see Supporting Table 1 and Supporting Figure 3 below) is evidence that ROIMCR could identify the “other” components present in the sample. We expected to detect additional F-containing compounds in the unknown gull-egg samples, but unfortunately this was not the case. Most studies reporting PFAS in biological samples report similar compounds as the ones we have herein identified.

UHPLC-qTOF – MS1 and MS2 analysis

Analysis was carried out by ultra-high-performance liquid chromatography (UPLC) coupled to a Bruker Impact II Q-TOF mass spectrometer. Parameters from the cold Apollo ion source were set as follow: negative electrospray ionization with a capillary at 2500 V, dry gas temperature at 200°C, drying gas flow at 8 L/min, nebulizer at 2 bars, and plate offset at 500 V. Q-TOF was tuned and calibrated with sodium formate using 14 *m/z* selected ions for mass error calculation. An accuracy of 0.1 ppm was achieved. This calibration was carried out at every injection to monitor and detect any change in the signal. Acquisition was done in full scan mode at a mass range from 30 to 1000 m/z. Resolving power was 60.000 at full width at half maximum (FWHM) at *m/z* 200. DIA was obtained using 6 eV energy for MS1 and 30 eV energy for MS2 using bbCID from Bruker technology.

A chromatographic column Phenomenex C18 Luna Omega (100 mm length × 2.1 mm inner diameter, 100 Å pore size, 1.6 µm particle size) was employed at 40ºC. The mobile phase consisted of (A) MeOH:ACN (80:20, v/v) buffered with 10 mM $NH_4Ac_{(aq)}$ and (B) 10 mM $NH_4Ac_{(aq)}$ aqueous solution. Initial conditions were 50% A and 50% B kept for 1 min, increased to 90% A in 9 min (2 min hold time) and reaching initial conditions in 6 min. Flow rate was set at 0.3 mL/min. The injection volume was 2 µL. An XBridge C18 column (50 mm × 4.6 mm, 3.5 µm particle size) was used as trap

column to remove background PFAS contribution from the mobile phase and LC tubing.

The Multivariate Curve Resolution Alternating Least Squares (MCR-ALS) method

This MCR-ALS optimization method (Tauler, R. Multivariate curve resolution applied to second order data, Chemom Intel Lab Syst., 1995, 30, 133–146: Jaumot, J.; de Juan, A.; Tauler, R. MCR-ALS GUI 2.0: New features and applications, Chemom Intel Lab Syst., 2015, 140, 1-12) can be described by the two following Equations 1 and 2.

C is estimated from $\min_{\hat{\mathbf{S}},\mathbf{cons}} \left\| \hat{\mathbf{D}}_{\mathbf{PCA}} - \hat{\mathbf{C}}\hat{\mathbf{S}}^{\mathbf{T}} \right\|$ Equation 1

and

$\mathbf{S}^{\mathrm{T}}$ is estimated from $\min_{\hat{\mathbf{C}},\mathbf{cons}} \left\| \hat{\mathbf{D}}_{\mathbf{PCA}} - \hat{\mathbf{C}}\hat{\mathbf{S}}^{\mathbf{T}} \right\|$ Equation 2

Factor matrices **C** and $\mathbf{S}^{\mathrm{T}}$ are obtained iteratively during the ALS optimization solving the two least squares equations, where $\hat{\mathbf{D}}_{\mathbf{PCA}}$ is the Principal Component Analysis (PCA, 27. Jolliffe, I.T. Principal Component Analysis. 2nd ed. 2002, Springer Verlag, Berlin, Germany) reproduced data matrix, which is used to filter experimental noise and to stabilize the calculations for the preselected number of components, N, of the model. $\hat{\mathbf{C}}$ and $\hat{\mathbf{S}}^{\mathbf{T}}$ are the current least squares estimated concentrations and spectra factor matrices. Both estimations are performed under a set of constraints, like non-negativity constraints in **C** and $\mathbf{S}^{\mathrm{T}}$ factor matrices, and the normalization of the resolved mass spectra in $\mathbf{S}^{\mathrm{T}}$ to have their maximum signal intensity equal to one. ALS optimization is started with a preselected number of components, N, and with an initial estimation of either **C** or $\mathbf{S}^{\mathrm{T}}$**.** The number of components can be estimated for instance by Singular Value Decomposition (Golub, G.; van Loan, C. Matrix Computations. Second ed. 1989. John Hopkins University Press, Baltimore, USA) or by PCA (Jolliffe, I.T. Principal Component Analysis. 2nd ed. 2002, Springer Verlag, Berlin, Germany). This number is then validated by checking the chemical reliability of the component profiles obtained in **C** or $\mathbf{S}^{\mathrm{T}}$. In order to speed up the calculations and avoid divergence, instead of random initial estimations of **C** or $\mathbf{S}^{\mathrm{T}}$, the N more different elution columns or the N most different spectral rows of the experimental data matrix **D** (as in the purest variables selection approach, (Windig,W.; Guilment, J., Interactive self-modeling mixture analysis. Anal. Chem. 1991. 63, 1425–1432), or also if available, from the previous knowledge of the system. Finally, ALS convergence is tested when the relative standard deviation of the change of the residuals between two consecutive

iterations is lower than a preselected threshold value, for instance of 0.1%. And the quality of the fit finally achieved can be measured with the amount of explained data variance in percentage according to Equation 3.

Variance explained (R^2, %) $$R^2 = 100\,\frac{\sum_{i=1}^{I}\sum_{j=1}^{J} d_{i,j}^2 - \sum_{i=1}^{I}\sum_{j=1}^{J} r_{i,j}^2}{\sum_{i=1}^{I}\sum_{j=1}^{J} d_{i,j}^2}$$ Equation 3

where $r_{i,j} = d_{i,j} - \hat{d}_{i,j}$

Explanation of Supporting Table 1 (below).

PFAS in the standard mixtures were identified using the m/z values of the higher intensity signals of the ROIMCR resolved MS1 and MS2 spectra and using the retention times of the resolved elution profiles (peak maxima). The estimated ROI m/z values of the (precursor) ions with the highest MS1 signal intensities were compared with the theoretical m/z values and their errors in ppm are given. In all cases, ppm errors of MS1 signals were low (< 6 ppm). *m/z* values of the major intensity signals of the MS2 spectra corresponding to every component resolved by the ROIMCR method are also given (in the last column) and further used for chemical confirmation of the different resolved components. Underlined *m/z* MS2 signals were coincident with the ones found using the target analysis of the Brucker instrument software. In addition, additional signals encountered by ROIMCR are given and compared to those from the target approach. In general, several additional MS2 signal ions were resolved by ROIMCR. The threshold value selected to consider these signals significant was 5% in and 20% of the maximum signal, depending on the case, and they were normalized to a maximum intensity value of 1 during MCR-ALS analysis. In general, there was a high level of coincidence, and the results obtained confirm that the proposed approach is adequate for the direct identification of the chemical compounds associated with the ROIMCR resolved components, using simultaneously the MS1 and MS2 ion signals from the data independent acquisition (DIA) system.

At the bottom of the Table, six components (35-40) were resolved by ROIMCR in the analysis of gull egg samples which were not present in the PFAS standard samples. The *m/z* values of their MS1 MS2 signals and the retention times of their elution profiles are given. These compounds were eluting at the end of the chromatogram (see also Supporting Figure 4). Two of these compounds (nr. 35 and 36) were identified (HMDB data base) as being palmitic acid and docosahexaenoic acid. The other four compounds gave well defined elution and spectra profiles (see Table values), but they could not be identified in the currently available PFAS databases.

Supporting Table 1 Identification of ROIMCR components.

ROIMCR Component [1]	PFAS compound[2]	Abbreviation[3]	Retention time (sec)[4]	Theoretical MS1 mz[5]	Experimental MS1 mz[6]	Error (ppm)[7]	MS2 signals[8]		Score[9]
							m/z	Rel. Intensity	
1	Perfluoro-n-butanoic acid	PFBA	116.4	212.9792	212.9797	2.35	168.9896<br>212.9789	999<br>332	9.9*
2	Perfluoro-n-[1,2,3,4-13C4]butanoic acid	MPFBA	116	216.9928	216.9936	3.69	171.9995	-	-
3	Perfluoro-n-pentanoic acid	PFPeA	161.7	262.976	262.9763	1.14	218.9864<br>262.9752	999<br>368	9.9*
4	Potassium perfluoro-1-butanesulfonate	L-PFBS	186.4	298.943	298.9433	1	79.9573<br>98.9554<br>298.9433	536<br>153<br>999	9.6*
5	Sodium,1H,2H,2H-perfluorohexanesulfonate	4:2 FTS	231.6	326.9743	326.9745	0.61	79.9573<br>80.9635<br>306.9686<br>326.9748<br>327.9775	77<br>178<br>188<br>999<br>75	8.1*
6	Perfluoro-n-hexanoic acid	PFHxA	258.4	312.9728	312.9727	0.32	118.9927<br>268.9833<br>269.9862	459<br>999<br>210	9.1*
7	Perfluoro-n-[1,2-13C2]hexanoic acid	MPFHxA	248	314.9804	314.9789	4.76	118.9927<br>119.9957<br>269.986	-	-

8	Sodium perfluoro-1-pentanesulfonate	L-PFPeS	301.6	348.9398	348.9403	1.43	348.9401	999	9.9**
9	Perfluoro-n-heptanoic acid	PFHpA	386.1	362.9696	362.9699	0.83	118.9927<br>168.9895<br>318.9802	387<br>744<br>999	9.2*
10	Potassium perfluorohexanesulfonate	br-PFHxS	412.8	398.9366	398.9369	0.75	398.937	999	9.9**
11	Sodium perfluoro-1-hexane[18O2]sulfonate	MPFHxS	404	402.9466	402.9443	5.71	400.9395<br>402.9441	-	-
12	Sodium 1H,1H,2H,2H-perfluorooctanesulfonate	6:2 FTS	462.2	426.9679	426.9675	0.94	80.9653<br>406.9627<br>426.9682<br>427.9717	136<br>219<br>999<br>82	9.2*
13	Perfluoro-n-octanoic acid	PFOA	476.6	412.9664	412.9664	0	118.9927<br>168.9895<br>218.9864<br>368.9771	139<br>999<br>221<br>947	9.3*
14	Perfluoro-n-[1,2,3,4-13C4]octanoic acid	MPFOA	468	416.9808	416.9792	3.83	168.9895<br>171.9995<br>371.9857	-	-
15	Sodium perfluoro-1-heptanesulfonate	L-PFHpS	495.2	448.9334	448.9335	0.22	448.9337	999	9.9**
16	Perfluoro-n-nonanoic acid	PFNA	548.7	462.9632	462.9636	0.86	168.9895<br>218.9864<br>418.9738	551<br>471<br>999	9.4*

17	Perfluoro-n-[1,2,3,4,5-13C5]nonanoic acid	MPFNA	540	467.98	467.9797	0.64	168.9895<br>171.9995<br>218.9868<br>422.9867<br>498.289	-	-
18	Potassium perfluorooctanesulfonate	br-PFOS	558.9	498.9302	498.9301	0.2	498.9299<br>499.9332<br>502.9435	999<br>83<br>130	8.2*
19	Sodiumperfluoro-1-[1,2,3,4-13C4]octanesulfonate	MPFOS	552	502.9436	502.9435	0.2	498.9299<br>502.9435	-	-
20	Sodium 1H,1H,2H,2H-perfluorodecanesulfonate	8:2 FTS	600.2	526.9615	526.9612	0.57	526.9615	999	9.9**
21	Perfluoro-n-decanoic acid	PFDA	606.3	512.96	512.9611	2.14	168.9895<br>218.9864<br>268.9833<br>468,9703<br>469.9735<br>512.9626	305<br>412<br>370<br>999<br>237<br>187	8.9*
22	Perfluoro-n-[1,2-13C2]decanoic acid	MPFDA	598	514.966	514.967	1.94	168.9895<br>169.9928<br>218.9864<br>219.9896<br>268.9833<br>269.9862<br>469.9735	-	-
23	Sodium perfluoro-1-nonanesulfonate	L-PFNS	614.6	548.927	548.9266	0.73	548.9264,<br>549.9308	999, 70	7.2*
24	N-methylperfluorooctanesulfonamidoacetic	br-N-	629	569.9673	569.9675	0.35	418.9738	999	9.9**

	acid	MeFOSAA							
25	Perfluoro-n-undecanoic acid	PFUdA	655.7	562.9568	562.9568	0	168.9895<br>218.9864<br>268.9833<br>318.9802<br>418.9738<br>518.9668<br>519.9713<br>525.9774<br>583.9828	365<br>340<br>340<br>229<br>370<br>999<br>184<br>213<br>243	7.5**
26	Perfluoro-1-octanesulfonamide	FOSA	651.6	497.9462	497.9461	0.2	77.969<br>497.946	299<br>999	9.4*
27	N-ethylperfluorooctanesulfonamidoacetic acid	br- N EtFOSAA	655.7	583.983	583.9829	0.17	-	-	-
28	Perfluoro-n-[1,2-13C2]undecanoic acid	MPFUdA	647	564.9624	564.9639	2.66	61.9883<br>168.9895<br>218.9864<br>268.9833<br>269.9862<br>318.9802<br>319.9834	-	-
29	Sodium perfluoro-1-decanesulfonate	L-PFDS	659.8	598.9238	598.9236	0.33	598.9233	999	8.6*

30	Perfluoro-n-dodecanoic acid	PFDoA	696.9	612.9537	612.9543	0.98	168.9895<br>218.9864<br>268.9833<br>318.9802<br>368.9771<br>568.9636<br>569.9674	345<br>198<br>262<br>300<br>161<br>999<br>308	9.6*
31	Perfluoro-n-[1,2-13C2]dodecanoic acid	MPFDoA	688	614.9598	614.9611	2.11	168.9894<br>268.9833<br>269.9862<br>318.9802<br>319.9834<br>569.9674	-	-
32	Perfluoro-n-tridecanoic acid	PFTrDA	731.9	662.9505	662.9507	0.3	618.9611<br>662.9507<br>699.9208	367<br>999<br>232	8.0*
33	Sodium perfluoro-1-dodecanesulfonate	L-PFDoS	731.9	689.9174	698.9172	1.02	-	-	
34	Perfluoro-n-tetradecanoic acid	PFTeDA	762.8	712.9473	712.9465	1.12	168.9894<br>218.9864<br>268.9832<br>329.2882<br>368.9771<br>418.9738<br>668.9585<br>669.9623<br>712.9490	256<br>189<br>158<br>165<br>155<br>109<br>999<br>130<br>176	9.5**

35	Palmitoleic acid	-	750	253.217	253.2177	3.95	44.9982<br>253.217<br>254.221<br>329.2882	117<br>999<br>172<br>93	8.6*
36	Docosahexaenoic acid	DHA	766.9	327.2327	327.2337	3.06	283.2439<br>372.2337	168<br>999	8.9*
37	Unknown compound	-	734	-	301.2177	-	301.2175	-	
38	Unknown compound	-	764.9	-	626.3473	-	283.2439,<br>327.2337,<br>528.3103,<br>552.3106	-	
39	Unknown compound	-	769	-	602.3472	-	279.2332,<br>303.2333,<br>504.3103,<br>528.3103	-	
40	Unknown compound	-	775.2	-	303.233	-	303.2333	-	

[1] Resolved ROIMCR component; [2] PFAS (1-34) and other (35-40) compounds; [3] PFAS compound abbreviation; [4] Experimental retention time at the peak maximum of the ROIMCR elution profile in sec; [5] Theoretical MS1 m/z value of the precursor ion; [6] ROIMCR MS1 m/z value of the precursor ion; [7] m/z deviation (error) between theoretical m/z and ROIMCR m/z values on ppm; [8] ROIMCR MS2 m/z values of the fragments of this compound. The relative intensity values are normalized to 999. Underlined MS2 signals were coincident with those found in previous literature for this compound; [9] Scores for annotation using MS2 spectrum. The scores have been normalized to 10. * calculated from MoNA https://mona.fiehnlab.ucdavis.edu/ and ** calculated from MassBank of North America, https://massbank.eu/MassBank/

ROIMCR Comp [1]	Compound identification[2]	Abbreviation[3]	RT (sec)[4]	MS1 mz[5]	Exp. MS1 mz[6]	Error (ppm)[7]	MS2 signals[8]	Score[9]
1	Perfluoro-n-butanoic acid	PFBA	116.4	212.9792	212.9797	2.35	168.9896, 212.9789	9.9*
2	Perfluoro-n-[1,2,3,4-13C4]butanoic acid	MPFBA	116	216.9928	216.9936	3.69	171.9995	-
3	Perfluoro-n-pentanoic acid	PFPeA	161.7	262.976	262.9763	1.14	218.9864, 262.9752	9.9*
4	Potassium perfluoro-1-butanesulfonate	L-PFBS	186.4	298.943	298.9433	1	79.9573, 98.9554, 298.9433	9.6*
5	Sodium,1H,2H,2H-perfluorohexanesulfonate	4:2 FTS	231.6	326.9743	326.9745	0.61	79.9573, 80.9635, 306.9686, 326.9748, 327.9775	8.1*
6	Perfluoro-n-hexanoic acid	PFHxA	258.4	312.9728	312.9727	0.32	118.9927, 268.9833, 269.9862	9.1*
7	Perfluoro-n-[1,2-13C2]hexanoic acid	MPFHxA	248	314.9804	314.9789	4.76	118.9927, 119.9957, 269.9862	-
8	Sodium perfluoro-1-pentanesulfonate	L-PFPeS	301.6	348.9398	348.9403	1.43	348.9401	9.9*
9	Perfluoro-n-heptanoic acid	PFHpA	386.1	362.9696	362.9699	0.83	118.9927, 168.9895, 318.9802	9.2*
10	Potassium perfluorohexanesulfonate	br-PFHxS	412.8	398.9366	398.9369	0.75	79.9573, 398.9370, 399.9400, 402.9441, (403.949)	9.9*
11	Sodium perfluoro-1-hexane[18O2]sulfonate	MPFHxS	404	402.9466	402.9443	5.71	(83.9664, 399.9404), 400.9395, 402.9441	-
12	Sodium 1H,1H,2H,2H-perfluorooctanesulfonate	6:2 FTS	462.2	426.9679	426.9675	0.94	80.9653, 406.9627, 426.9682, 427.9717	9.2*
13	Perfluoro-n-octanoic acid	PFOA	476.6	412.9664	412.9664	0	118.9927, 168.9895, 218.9864,	9.3*

							368.9771	
14	Perfluoro-n-[1,2,3,4-13C4]octanoic acid	MPFOA	468	416.9808	416.9792	3.83	168.9895, 171.9995, (218.9868), 371.9857	-
15	Sodium perfluoro-1-heptanesulfonate	L-PFHpS	495.2	448.9334	448.9335	0.22	(79.9574, 188.9396), 448.9337, 449.9376	9.9**
16	Perfluoro-n-nonanoic acid	PFNA	548.7	462.9632	462.9636	0.86	168.9895, 218.9864, (268.9832), 418.9738	9.3*
17	Perfluoro-n-[1,2,3,4,5-13C5]nonanoic acid	MPFNA	540	467.9800	467.9797	0.64	168.9895, 171.9995, 218.9868, (222.9993, 269.9859), 422.9867, 498.289	-
18	Potassium perfluorooctanesulfonate	br-PFOS	558.9	498.9302	498.9301	0.2	(79.9574, 188.9396), 498.9299, 499.9332, 502.9435	8.2*
19	Sodiumperfluoro-1-[1,2,3,4-13C4]octanesulfonate	MPFOS	552	502.9436	502.9435	0.2	(79.9574, 188.9396), 498.9299, 502.9435	-
20	Sodium 1H,1H,2H,2H-perfluorodecanesulfonate	8:2 FTS	600.2	526.9615	526.9612	0.57	526.9615	9.9**
21	Perfluoro-n-decanoic acid	PFDA	606.3	512.96	512.9611	2.14	168.9895, 218.9864, 268.9833, 468,9703, 469.9735, 512.9626	8.9*
22	Perfluoro-n-[1,2-13C2]decanoic acid	MPFDA	598	514.9660	514.967	1.94	168.9895, 169.9928, 218.9864, 219.9896, 268.9833, 269.9862, 469.9735	-
23	Sodium perfluoro-1-nonanesulfonate	L-PFNS	614.6	548.927	548.9266	0.73	(265.1472), 548.9264, 549.9308	7.2*
24	N-ethylperfluorooctanesulfonamidoacetic	br-N-MeFOSAA	629	569.9673	569.9675	0.35	418.9738	9.9**

	acid							
25	Perfluoro-1-octanesulfonamide	FOSA	651.6	497.9462	497.9461	0.2	77.969, (188.9386), 497.946	9.4*
26	Perfluoro-n-undecanoic acid	PFUdA	655.7	562.9568	562.9568	0	(118.9927), 168.9895, 218.9864, 268.9833, 318.9802, 418.9738, 518.9668, 519.9713, 525.9774, 583.9828	7.5**
27	Perfluoro-n-[1,2-13C2]undecanoic acid	MPFUdA	657	564.9624	564.9639	2.66	61.9883, (118.9927), 168.9895, (169.992), 218.9864, 268.9833, 269.9862, 318.9802, 319.9834 (418.972, 519.9694)	
28	N-ethylperfluorooctanesulfonamidoacetic acid	br- N EtFOSAA	655.7	583.983	583.9829	0.17	-	-
29	Sodium perfluoro-1-decanesulfonate	L-PFDS	659.8	598.9238	598.9236	0.33	598.9233, 599.9271	-
30	Perfluoro-n-dodecanoic acid	PFDoA	696.9	612.9537	612.9543	0.98	(118.9927), 168.9895, 218.9864, 268.9833, 318.9802, 368.9771, (418.972), 568.9636, 569.9674	8.6*
31	Perfluoro-n-[1,2-13C2]dodecanoic acid	MPFDoA	688	614.9598	614.9611	2.11	(118.9927), 168.9894, (169.992, 219.989), 268.9833, 269.9862, 318.9802, 319.9834, (418.972), 569.9674	9.6*
32	Perfluoro-n-tridecanoic acid	PFTrDA	731.9	662.9505	662.9507	0.3	618.9604, 698.9174, 699.9209	-
33	Sodium perfluoro-1-dodecanesulfonate	L-PFDoS	731.9	689.9174	698.9172	1.02	-	7.9*

34	Perfluoro-n-tetradecanoic acid	PFTeDA	762.8	712.9473	712.9465	1.12	168.9894, 218.9864, 268.9832, (318.9802), 329.2882, 368.9771, 418.9738, 668.9585, 669.9623, 712.9490	
35	Palmitoleic acid	-	750	253.217	253.2177	3.95	253.217, 254.221	9.5**
36	Docosahexaenoic acid	DHA	766.9	327.2327	327.2337	3.06	283.2439, 372.2337	8.6*
37	Unknown compound	-	734	-	301.2177	-	301.2175	8.9*
38	Unknown compound	-	764.9	-	626.3473	-	283.2439, 327.2337, 528.3103, 552.3106	
39	Unknown compound	-	769.0	-	602.3472	-	279.2332, 303.2333, 504.3103, 528.3103	
40	Unknown compound	-	775.2	-	303.233	-	303.2333	

[1] Resolved ROIMCR component; [2] PFAS (1-34) and other (35-40) compounds; [3] PFAS compound abbreviation; [4] Experimental retention time at the peak maximum of the ROIMCR elution profile in seg; [5] Theoretical MS1 m/z value of the precursor ion; [6] ROIMCR MS1 m/z value of the precursor ion; [7] m/z deviation (error) between theoretical m/z and ROIMCR m/z values on ppm; [8] ROIMCR MS2 m/z values of the fragments of this compound. Underlined MS2 signals were coincident with those found in previous literature for this compound; [9]ROIMCR MS2 spectra similarity (correlation) scores normalized to 10, according to * MoNA, MassBank of North America, https://mona.fiehnlab.ucdavis.edu/ and **MassBank, https://massbank.eu/MassBank/

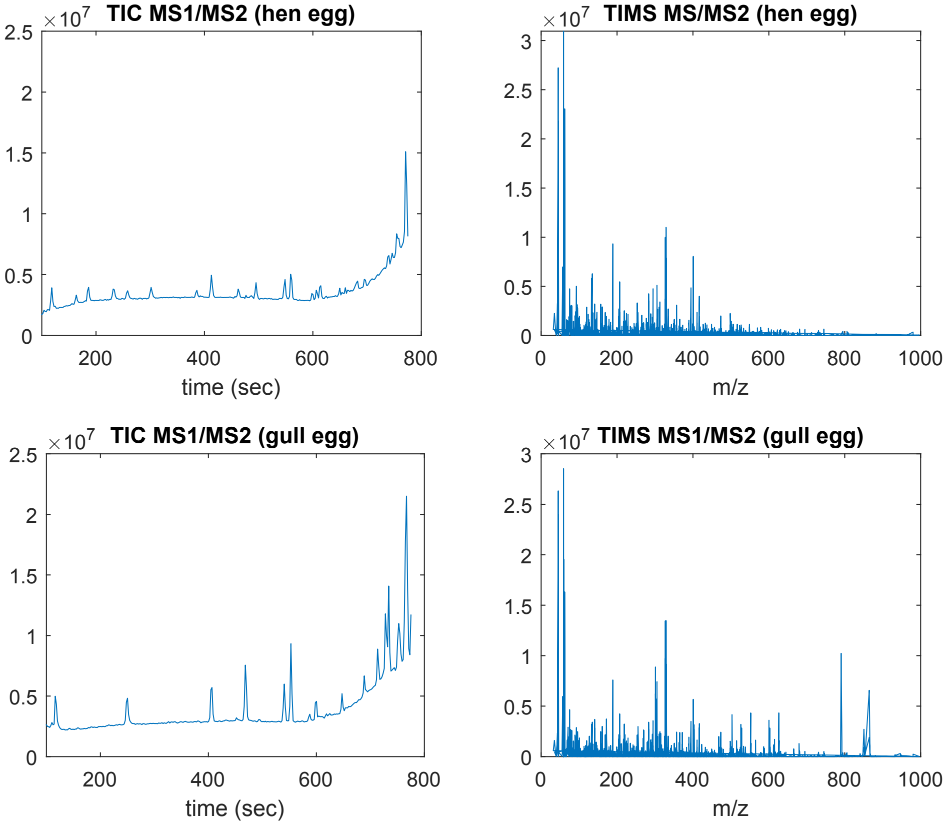

Supporting Figure 1 MS1/MS2 Sum Total Ion Chromatogram (TIC, left) and MS1/MS2 Sum Total Ion Mass Spectra (TIMS, right) of one hen egg (upper panels) and one gull egg (down panels) samples.

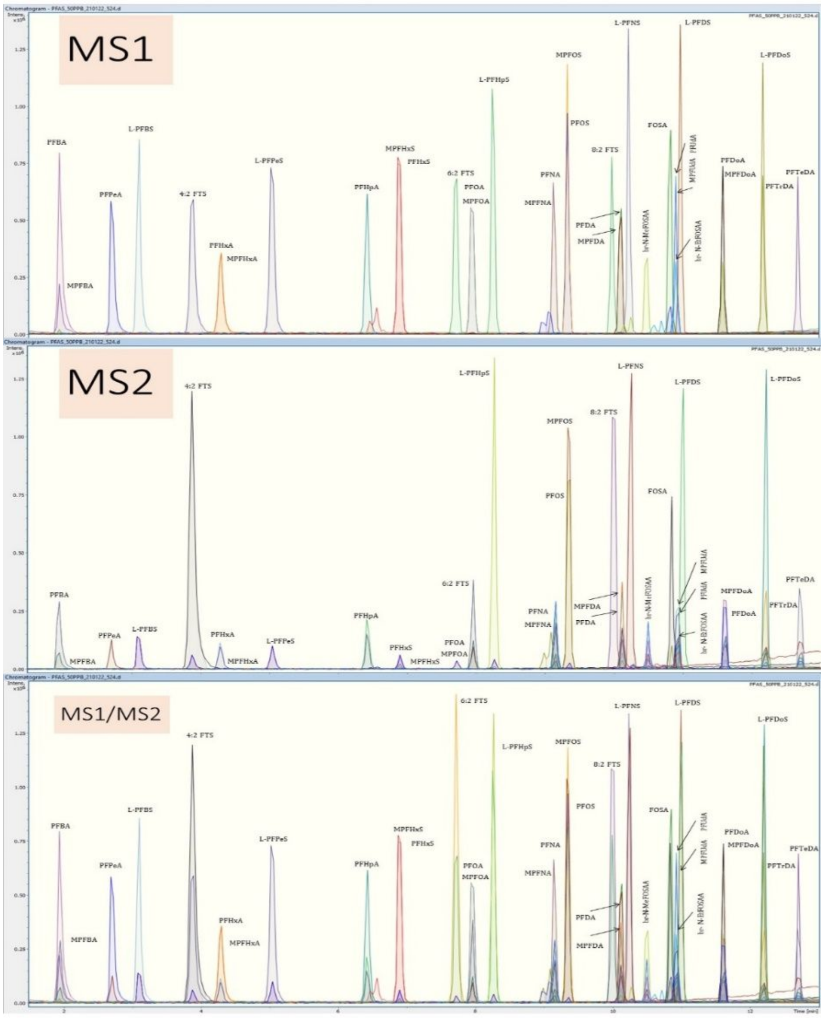

Supporting Figure 2 Elution profiles obtained by Compass Data Analysis (Bruker Scientific) of the 50 ng/mL standard PFAS mixture sample by MS1 (upper), MS2 (middle) and MS1/MS2 DIA (lower).

Exact *m/z* values for the precursor MS1 ions of all the PFAS present in the standard mixtures were first calculated. These *m/z* values were then searched in the experimental low energy MS1 chromatograms using the Compass Data Analysis (Bruker Scientific LLC. 2019, GmbH. Bremen, Germany) instrument software. During the *m/z* ions searching, an instrumental deviation of 0.5 Da was allowed. Differences between the theoretical and the experimental m/z values of the detected PFAS were always below 0.01 Da units. MS2 DIA fragment ions of every previously identified precursor ion were then searched by the instrument software at the same retention time using also previous knowledge on the fragmentation patterns of PFAS. In the upper part of the Figure 3, the elution profiles obtained by the data analysis software of the precursor ions in MS1 (top), MS 2 (middle) and both MS1 and MS2 ions (bottom) are given. Results shown in this Supporting Figure 3 for the target analysis approach are coincident with those obtained by the non-target ROIMCR analysis approach proposed in this work shown in Figure 3 of the main manuscript.

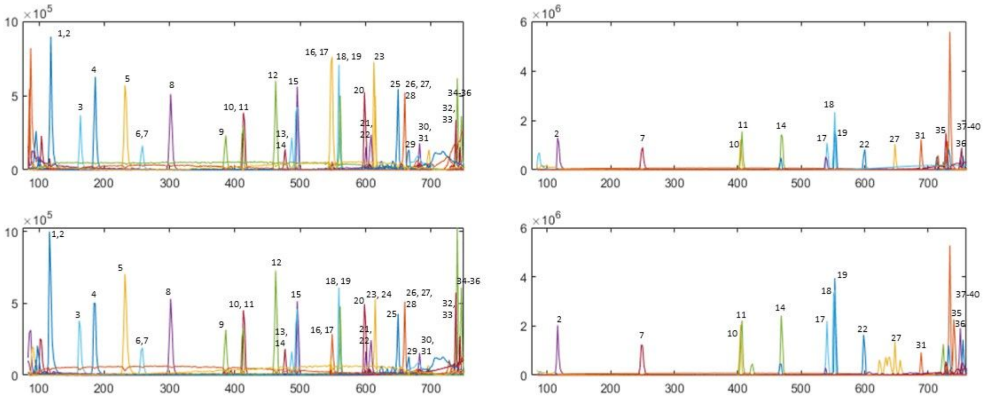

Supporting Figure 3. ROIMCR results in the analysis of two hen egg (on the left) and two gull egg samples (on the right) by MS1/MS2 DIA.

Numbers in the Figures give the compound identification according to annotation of PFAS in Table1 and Figure 3 of the manuscript, and in Supporting Table 1 (see above). Two plots on the left are ROIMCR results for the two hen egg samples, and two plots on the right are ROIMCR results for *Larus audouinii* and *Larus michahellis* gull egg samples. Compounds in the mass-labelled surrogates (2, 7, 11, 14, 17, 19, 22, 27 and 31) were resolved in the two gull egg samples. Compounds 35 (palmitoleic acid) and 36 (docosahexaenoic acid) were present in hen and gull egg samples and were identified as palmitoleic and docosahexaenoic acids. Compounds 37-40 were only present in gull-egg samples and could not be identified.
